# A New Look at Calendar Anomalies: Multifractality and Day-of-the-Week Effect

**DOI:** 10.3390/e24040562

**Published:** 2022-04-17

**Authors:** Darko Stosic, Dusan Stosic, Irena Vodenska, H. Eugene Stanley, Tatijana Stosic

**Affiliations:** 1 Centro de Informática, Universidade Federal de Pernambuco, Av. Luiz Freire s/n, Recife 50670-901, PE, Brazil; dd.stosic@gmail.com (D.S.); dbstosic@bu.edu (D.S.); 2 Department of Administrative Sciences, Metropolitan College, Boston University, 1010 Commonwealth Avenue, Boston, MA 02215, USA; 3 Center for Polymer Studies, Department of Physics, Boston University, 590 Commonwealth Avenue, Boston, MA 02215, USA; hes@bu.edu; 4 Departamento de Estatística e Informática, Universidade Federal Rural de Pernambuco, Rua Dom Manoel de Medeiros s/n, Dois Irmãos, Recife 52171-900, PE, Brazil; tastosic@gmail.com

**Keywords:** calendar anomalies, day-of-the-week effect, market indices, multifractal detrended fluctuation analysis

## Abstract

Stock markets can become inefficient due to calendar anomalies known as the day-of-the-week effect. Calendar anomalies are well known in the financial literature, but the phenomena remain to be explored in econophysics. This paper uses multifractal analysis to evaluate if the temporal dynamics of market returns also exhibit calendar anomalies such as day-of-the-week effects. We apply multifractal detrended fluctuation analysis (MF-DFA) to the daily returns of market indices worldwide for each day of the week. Our results indicate that distinct multifractal properties characterize individual days of the week. Monday returns tend to exhibit more persistent behavior and richer multifractal structures than other day-resolved returns. Shuffling the series reveals that multifractality arises from a broad probability density function and long-term correlations. The time-dependent multifractal analysis shows that the Monday returns’ multifractal spectra are much wider than those of other days. This behavior is especially persistent during financial crises. The presence of day-of-the-week effects in multifractal dynamics of market returns motivates further research on calendar anomalies for distinct market regimes.

## 1. Introduction

Market prices should incorporate and reflect all available information at any point in time, according to the Efficient Market Hypothesis (EMH) [1,2]. Yet, various studies [3,4,5,6] show that financial markets often become inefficient, and their behavior no longer follows that of a random walk. Stock markets can instead deviate from the rules of the EMH in the form of anomalies. Anomalies can be broadly categorized into calendar, fundamental and technical anomalies [7]. The most studied set of pricing anomalies is calendar or seasonal anomalies that represent systematic patterns of security returns around certain calendar points. Calendar anomalies include the day-of-the-week effect [8,9,10,11], turn-of-the-month effect [12,13,14,15], turn-of-the-year effect [16,17,18,19] and holiday effect [20,21,22,23]. The day-of-the-week effect refers to the tendency of stocks to exhibit significantly higher returns on one particular day compared with other days of the week. Cross [24] first provided evidence of day-of-the-week effects on the Standard and Poor’s index, reporting that price returns are significantly negative on Mondays. Since then, this phenomenon has been extensively studied and discovered in other financial markets such as specific equity markets [25,26,27], exchange rates [28,29], fixed-income securities [30], crude oil [31], gold [32] and cryptocurrencies [33]. For a detailed review of seasonal anomalies, please see [34,35].

Financial markets have attracted much attention from researchers in related fields such as econophysics, paving the road for new perspectives and understanding of financial markets by drawing concepts from statistical physics such as fractals and multifractals [36,37,38,39], information theory [40,41] and network structures [42,43,44] (see [45] and the references therein for a comprehensive review). While many well-known conclusions in the literature on an array of financial markets (including market indices, stocks, exchange rates and commodities) can be attributed to econophysics, there are still a number of important phenomena to be investigated from this perspective. To the best of our knowledge, one such phenomenon that remains to be unearthed is the calendar anomaly, and our study makes a contribution in this direction.

In this paper, we use multifractal analysis to evaluate if the temporal dynamics of market returns exhibit calendar anomalies such as day-of-the-week effects. We apply multifractal detrended fluctuation analysis (MF-DFA) [46] to the daily returns of market indices around the world for each day of the week (Monday returns, Tuesday returns and so on). We then compare the multifractal parameters, the position of maximum width and asymmetry of the multifractal spectrum, which quantify long-term correlations, the degree of multifractality and the dominance of large or small fluctuations in the return series for each day of the week. The economic literature states that market practitioners have been aware of the Monday effect as early as the 1920s [47]. For some markets, this effect disappears as the market becomes more efficient [48,49]. Other studies offer insight into the Monday effect being more prominent toward the end of the month [50] and during periods dominated by bad news [51]. To observe this behavior over time, we perform time-dependent multifractal analysis on the United States (GSPC) market by calculating the multifractal spectra of the return series in a sliding window. This computationally intensive and relatively novel approach, which has been implemented in only a few studies [52,53,54], permits us to analyze the temporal evolution of multifractal parameters which are related to different properties of market fluctuation, leading to better understanding of the underlying stochastic processes. The rest of this paper is organized as follows. Section 2 introduces the MF-DFA and the time-dependent methods. Section 3 describes the market data. Section 4 presents the results, and Section 5 draws the conclusion.

## 2. Methods

While fractal processes are characterized by long-term correlations that are described by a single scaling exponent, multifractal time series subsets with small and large fluctuations can scale differently, and the analysis of long-term correlations results in a hierarchy of scaling exponents [46]. Multifractal analysis of temporal series can be performed using different methods, such as the wavelet transform modulus maxima (WTMM) method [55], multifractal detrended fluctuation analysis (MF-DFA) method [46] and multifractal detrending moving average method (MF-DMA) [56]. In this work, we employ MF-DFA, which has been found to produce reliable results [57] and has been widely used to analyze physiological signals [58,59,60], geophysical data [61], weather data [62], and financial time series [63].

The implementation of the MF-DFA algorithm can be described as follows [46]:

i The first step is the integration of the original series x(i),i=1,…,N to produce
  (1) $$\begin{array}{c} X\left(k\right)=\sum_{i=1}^{k}\left[x\left(i\right)-\langle x\rangle \right],\;k=1,\ldots ,N, \end{array}$$
  where $\langle x\rangle =\frac{1}{N}\sum_{i=1}^{k}x\left(i\right)$ is the average.
ii Next, the integrated series X(k) is divided into $N_{n}=int\left(N/n\right)$ non-overlapping segments of a length *n*, and in each segment $\nu =1,\ldots ,N_{n}$, the local trend $X_{n,\nu }\left(k\right)$ is estimated as a linear or higher order polynomial least square fit and subtracted from X(k).
iii The detrended variance
  (2) $$\begin{array}{c} F^{2}\left(n,\nu \right)=\frac{1}{n}\sum_{k=(\nu -1)n+1}^{\nu n}{\left[X\left(k\right)-X_{n,\nu }\left(k\right)\right]}^{2} \end{array}$$
  is calculated for each segment and then averaged over all segments to obtain the *q*th order fluctuation function:
  (3) $$\begin{array}{c} F_{q}\left(n\right)={\left\{\frac{1}{N_{n}}\sum_{\nu =1}^{N_{n}}{\left[F^{2}\left(n,\nu \right)\right]}^{q/2}\right\}}^{1/q}, \end{array}$$
  where, in general, *q* can take on any real value except zero.
iv Repeating this calculation for all box sizes provides the relationship between the fluctuation function $F_{q}\left(n\right)$ and box size *n*. $F_{q}\left(n\right)$ increases with *n* according to a power law $F_{q}\left(n\right)∼n^{h(q)}$ if long-term correlations are present. The scaling exponent h(q) is obtained as the slope of the linear regression of $\log F_{q}\left(n\right)$ versus logn.

The power law exponent h(q) is called the generalized Hurst exponent, where for stationary time series, h(2) is identical to the well-known Hurst exponent *H*. For positive *q* values, h(q) describes the scaling behavior of large fluctuations, while for negative *q* values, h(q) describes the scaling behavior of small fluctuations, while h(q) is independent of *q* for monofractal time series and a decreasing function of *q* for multifractal time series.

The generalized Hurst exponents are related to the Renyi exponents τ(q) defined by the standard partition function-based multifractal formalism τ(q)=qh(q)−1. For the monofractal signals, τ(q) is a linear function of *q* (as h(q)=const.) and for multifractal signals τ(q) is a nonlinear function of *q*. A multifractal process can also be characterized by the singularity spectrum f(α), which is related to τ(q) through the Legendre transform:

(4) $$\begin{array}{c} \alpha \left(q\right)=\frac{d\tau (q)}{dq}, \end{array}$$

(5) $$\begin{array}{c} f(\alpha (q))=q\alpha (q)-\tau (q), \end{array}$$

where f(α) is the fractal dimension of the support of singularities in the measure with Lipschitz–Holder exponent α. The singularity spectrum of the monofractal signal is represented by a single point in the f(α) plane, whereas the multifractal process yields a single humped function.

Multifractal spectra reflect the level of complexity of the underlying stochastic process and can be characterized by a set of three parameters, which are determined as follows. The singularity spectra are fitted to a fourth degree polynomial:

(6) $$\begin{array}{c} f\left(\alpha \right)=A+B\left(\alpha -\alpha_{0}\right)+C{\left(\alpha -\alpha_{0}\right)}^{2}+D{\left(\alpha -\alpha_{0}\right)}^{3}+E{\left(\alpha -\alpha_{0}\right)}^{4} \end{array}$$

The multifractal spectrum parameters are found as the position of the maximum $\alpha_{0}={arg max}_{\alpha }f\left(\alpha \right)$, the width of the spectrum $W=\alpha_{max}-\alpha_{min}$ obtained from extrapolating the fitted curve to zero, and the skew parameter $r=\left(\alpha_{max}-\alpha_{0}\right)/\left(\alpha_{0}-\alpha_{min}\right)$, where r=1 for symmetric shapes, r>1 for right-skewed shapes and r<1 for left-skewed shapes. These three parameters can be used to evaluate the complexity of the underlying process. A small value of $\alpha_{0}$ means that the process is correlated and more regular in appearance. The width *W* of the spectrum measures the degree of multifractality of the process, where a wider range of fractal exponents leads to “richer” structures. The skew parameter *r* indicates which fractal exponents are dominant: the f(α) spectrum is right-skewed (r>1), and the process is characterized by a “fine structure” (small fluctuations) if high fractal exponents are dominant, whereas the process is more regular or smooth, the f(α) spectrum is left-skewed (r<1), and the fractal exponents describe the scaling of large fluctuations if the low fractal exponents are dominant. In summary, a signal with a high value of $\alpha_{0}$, a wide range *W* of fractal exponents (higher degree of multifractality) and a right-skewed shape (r>1) may be considered more complex than one with the opposite characteristics [60].

The two sources of multifratality in a time series are (1) a broad probability density function for the values of the time series and (2) different long-term correlations for small and large fluctuations. The type of multifractal can be found by randomly shuffling the series and analyzing its behavior. For multifractals of the second type, the shuffled series exhibits simple random behavior (since long-term correlations are destroyed), and the width of the f(α) spectrum is reduced to a single point. For multifractals of the first type, the width of the f(α) spectrum remains the same (since the probability density cannot be removed), and for multifractals of types 1 and 2, the shuffled series shows weaker multifractality than the original series [46].

The time-dependent MF-DFA algorithm is based on the sliding window technique and yields a temporal evolution of multifractality in the system. Given a time series $x=x_{1},\ldots ,x_{N}$, many sliding windows $z_{t}=x_{1+t\Delta },\ldots ,x_{w+t\Delta },t=0,1,\ldots ,\left[\frac{N-w}{\Delta }\right]$ are constructed, where w≤N is the window size, Δ≤w is the sliding step and the operator [.] denotes taking the integer part of the argument. The values of the time series in each window $z_{t}$ are then used to calculate the multifractal spectrum at a given time *t* using the method described above. This allowed us to obtain time evolutions for the three complexity parameters.

## 3. Data

**Figure 1:**
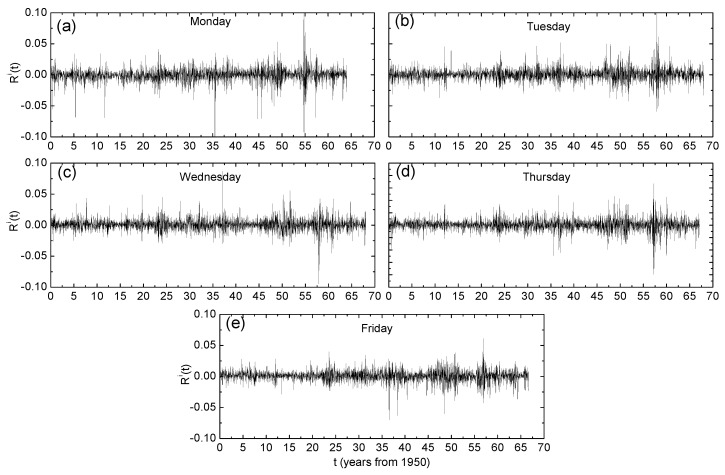
Time series for (**a**) Monday, (**b**) Tuesday, (**c**) Wednesday, (**d**) Thursday and (**e**) Friday day-resolved price returns $R^{i}$ of the United States (GSPC) market index.

We analyzed the time series of 19 major stock market indices that appear on the website https://finance.yahoo.com/world-indices/ (accessed on 2 January 2022), which are listed in Table 1. The period under study spanned the earliest recorded date for each index up to the end of 2018. For each of the market indices with consecutive workday closing price values S(t),t=1,…,N, we calculated the daily logarithmic returns:

(7) $$\begin{array}{c} R_{t}\equiv \ln\frac{S(t)}{S(t-1)}\;t=2,\ldots ,N, \end{array}$$

where the returns for Monday were calculated using the closing price of the previous Friday, while for other days of the week, two consecutive workday closing price values were used. Next, we constructed time series from the returns $R_{t}$ for each day of the week (Monday returns, Tuesday returns and so on):

(8) $$\begin{array}{c} R^{i}=\left\{R_{t_{i}},R_{t_{i}+5},\ldots ,R_{t_{i}+5\left[\frac{N}{5}\right]}\right\}, \end{array}$$

where i=1,…,5 denotes the index of the weekday, $R_{t_{i}}$ corresponds to the first occurrence of day *i* in the returns series $R_{t},t=2,\ldots ,N$ and the operator [.] denotes taking the integer part of the argument. Figure 1 reveals that the fluctuations in the returns varied between different days. While Monday exhibited the most pronounced negative returns, the fluctuations for other days dominated at specific time intervals. This is a well-known day-of-the-week effect which was found for the US market [8,25].

The MF-DFA method was applied to the day-resolved returns $R^{i}$ of major stock market indices, where local trends were fitted with a second-degree polynomial m=2. Next, we performed a fourth-order polynomial regression on the singularity spectra f(α) to determine the position of the maximum $\alpha_{0}$ and the zeros of the polynomial $\alpha_{\max}$ and $\alpha_{\min}$. From the polynomial fits, we calculated three measures of complexity: the position of the maximum $\alpha_{0}$, the width of the spectrum $W=\alpha_{\max}-\alpha_{\min}$ and the skew parameter $r=\left(\alpha_{\max}-\alpha_{0}\right)/\left(\alpha_{0}-\alpha_{\min}\right)$. These parameters were then used to determine the multifractal behavior of the day-resolved price returns.

## 4. Results

### 4.1. Day-of-the-Week Effect

**Figure 2:**
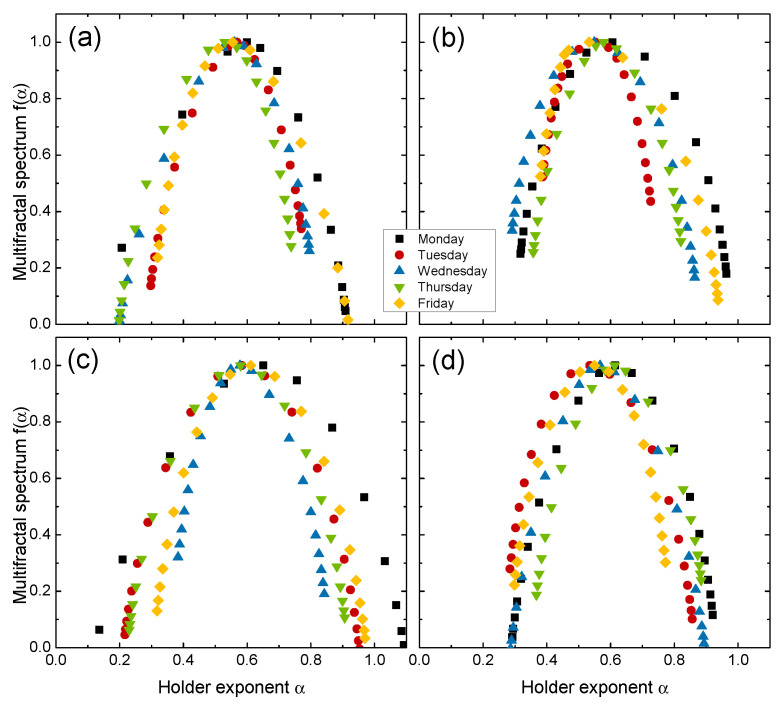
Multifractal spectrum f(α) for day-resolved price returns $R^{i}$ of (**a**) the United States (GSPC), (**b**) South Korea (KS11), (**c**) Chile (IPSA) and (**d**) France (FCHI) market indices.

Complexity measures derived from the singularity spectra were used to study the multifractal behavior of the price returns for every day of the week. We first considered multifractality in the day-resolved price returns from four distinct markets: the United States (GSPC), South Korea (KS11), Chile (IPSA) and France (FCHI). The multifractal spectra for each day using the four markets are illustrated in Figure 2. We observed that the day-of-the-week effects led to significant differences in multifractal behavior: (1) the positions of the maxima $\alpha_{0}$ were shifted to the right $\left(\alpha_{0}>0.5\right)$ for the Monday returns, and (2) the spectrum widths *W* were wider on Monday than those for returns from other days. There seemed to be no consistent differences in the skew parameter *r*, which implies that both large and small fluctuations are present for different days of the week (e.g., see Table 2). These results indicate that the Monday returns exhibited more persistent behavior and richer multifractal structures, which led to more complex time series than other day’s returns. Our findings are consistent with results obtained from [25], which indicated that Monday had the largest anomalies (day-of-the-week effect) because of the weekend gap in trading hours. Other days of the week did not exhibit any visible patterns in multifractal behavior for either the position or width of the spectrum.

**Figure 3:**
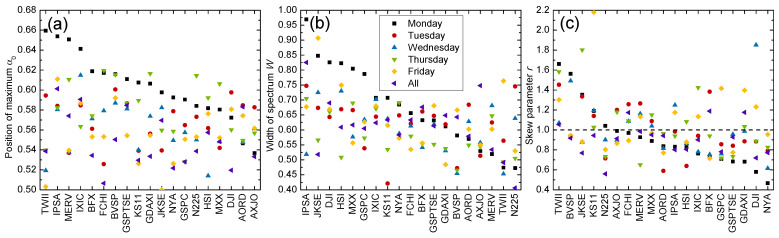
Complexity parameters (**a**) position of maximum $\alpha_{0}$, (**b**) spectrum width *W*, and (**c**) skew parameter *r*, for day-resolved price returns of the market indices listed in Table 1, sorted from largest to smallest.

We expanded our investigation to other markets listed in Table 1. Figure 3 reveals that the multifractal spectra of the Monday returns were dominantly right-shifted $\left(\alpha_{0}>0.5\right)$ compared with other days for most analyzed markets. Notable exceptions included the United States (DJI), Australia (AORD, AXJO), where the Tuesday returns were more persistent, and Japan (N225), where the Thursday returns exhibited stronger persistency. The width of the multifractal spectrum displayed similar tendencies to its position, where the Monday returns possessed broader multifractal widths. Yet, we found that more markets tended to have other days with richer multifractal structures; the multifractal spectra were the widest for the Friday returns in Taiwan (TWII) and the Tuesday returns in Japan (N225) and Australia (AORD), as opposed to the markets with dominant Monday returns considered so far. It has been noted that the day-of-the-week effect occurs on different distinct days of the week for different markets [25]. Considering both parameters $\alpha_{0}$ and *W*, we observed that the North American, European and some Asian (South Korea, Indonesia and Hong Kong) and Latin American markets (Chile and Mexico) tended to show both stronger persistency and stronger multifractality for the Monday returns, while for Australia, Indonesia and Taiwan, this tendency was found for the Tuesday returns. This is also in agreement with the literature, where it was found that some Asian markets displayed a Tuesday anomaly, which is one day out of phase with North American markets due to different time zones [64]. Patterns in the skew of multifractal spectra for a given day of the week are again hard to discern across distinct markets, where both small and large fluctuations exist. Values of the multifractal complexity parameter are listed in Table 2. Our results indicate that while most markets exhibit more complex behavior for Monday returns, some markets have other days with largest anomalies (day-of-the-week effect) such as Tuesday, Thursday and Friday returns. This is expected from literature where it was found that different day-of-the-week effects exist for different markets [25].

### 4.2. Comparison to Bulk Behavior

The day-resolved multifractal spectra could also be compared to those for the whole time series. The motivation for such a comparison is to provide more insight on the relation between multifractality and the day-of-the-week effect. From Figure 3, we found that many markets (IPSA, KS11, GSPTSE and MMX) exhibited distinct multifractal properties for a particular day (e.g., Monday returns), while the whole series displayed similar multifractal behavior to the bulk, or the remaining days of the week. For other markets (DJI, AXJI and N225), the overall multifractality of the series differed widely from the multifractal spectrum for each day of the week. This suggests that the day-of-the-week effects resulted in different multifractalities for these markets. We could further classify the markets into one of two multifractal behaviors: (1) bulk multifractality, which only differs for one particular day of the week, and (2) day-of-the-week multifractality, which is unique to every day and differs from the bulk behavior.

### 4.3. Source of Multifractality

**Figure 4:**
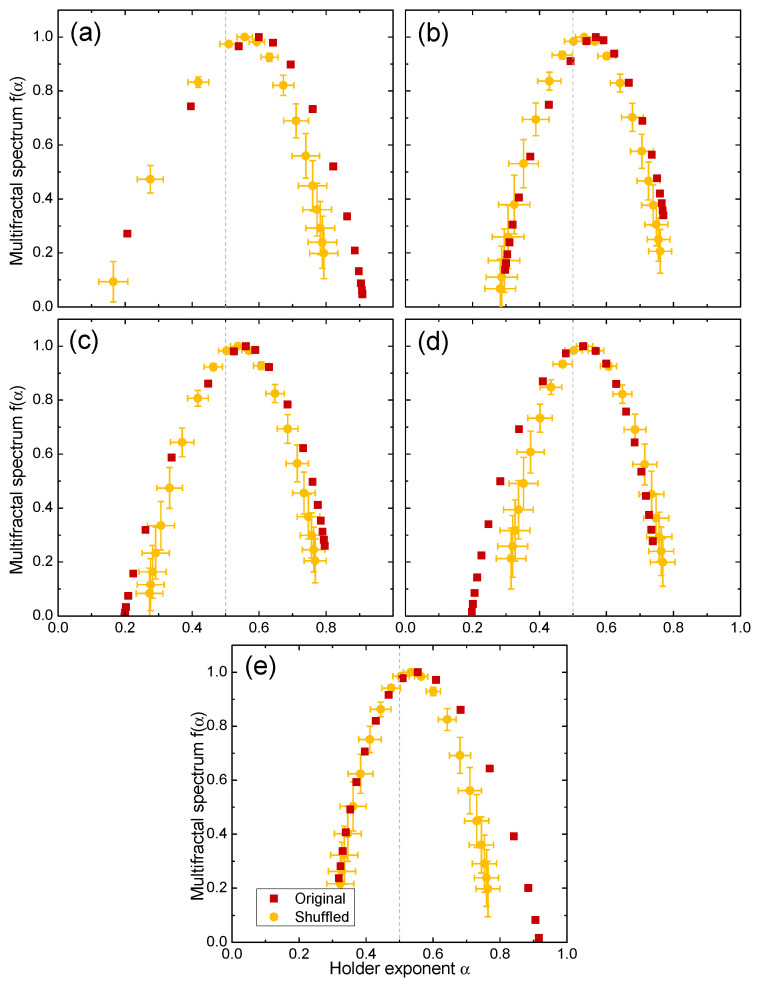
Original and shuffled multifractal spectra f(α) for (**a**) Monday, (**b**) Tuesday, (**c**) Wednesday, (**d**) Thursday and (**e**) Friday day-resolved price returns of the United States (GSPC) market.

We shuffled the time series of the day-resolved returns for the four markets and then applied MF-DFA to determine the source of multifractality. The shuffling procedure performed 1000×N transpositions on each series and was repeated 100 times with different random number generator seeds in order to obtain statistics such as the mean and standard deviation. Figure 4 reveals that for the United States (GSPC), the right-hand side of the spectrum (effect of small fluctuations) was mildly affected by shuffling on Mondays and Fridays, while the left side of the spectrum (effect of large fluctuations) was affected primarily on Thursdays (and less so on Wednesdays), and the position remained the same for all of the day-resolved returns. This indicates that multifractality arose primarily from a broad probability density function [65], and the long-term correlations had only a minor impact on some days of the week.

While it may be argued that destroying correlations by shuffling leads to strictly monofractal behavior and leaving only finite size effects, as shown for the qGaussian distributions using MFDFA [66] and market volatility data using partition function formalism [67], in the current case, shuffling left the spectrum width only slightly narrowed down, in agreement with previous MFDFA studies of market returns [65]. Even if upon shuffling only a finite size effect remained, different effects on different days of the week on small and large fluctuations provided novel insight into the market behavior.

Table 3 lists the changes in spectra position ($\Delta \alpha_{0}$) and width (ΔW) after shuffling the day-resolved returns for GSPC, KS11, IPSA and FCHI. We found that the Monday returns tended to exhibit the strongest effect from shuffling, where aside from the probability density function, long-term correlations also contributed to multifractality.

### 4.4. Time Evolution

**Figure 5:**
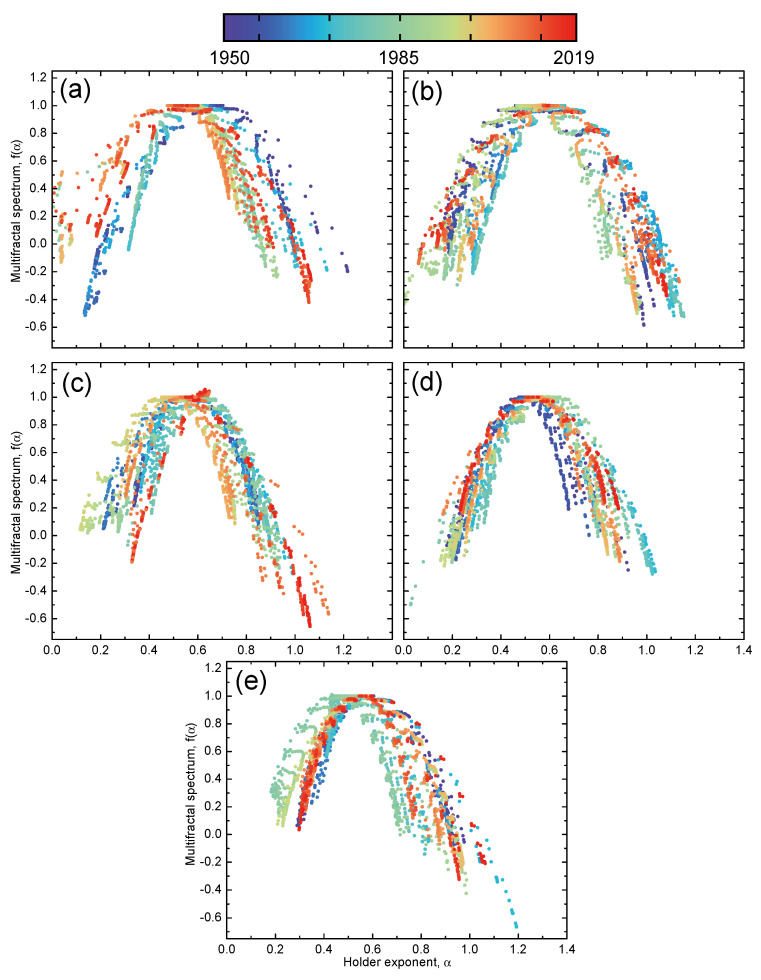
Time evolution of the multifractal spectrum f(α) for (**a**) Monday, (**b**) Tuesday, (**c**) Wednesday, (**d**) Thursday and (**e**) Friday day-resolved price returns of the United States (GSPC) market. A sliding window of 14 years and monthly intervals were used for the period spanning from 1950 to 2019.

**Figure 6:**
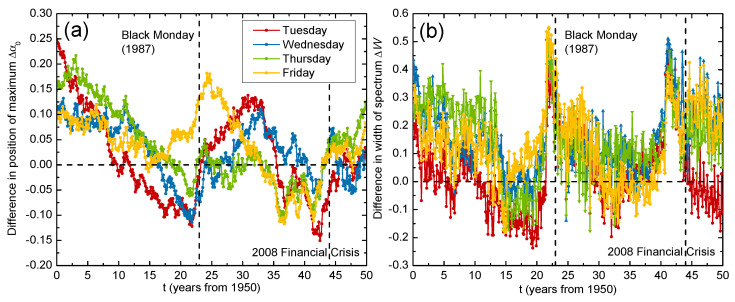
Time evolution of differences in complexity parameters (**a**) $\alpha_{0}$ and (**b**) *W* derived from the multifractal spectra f(α) between Monday and other day-resolved price returns for the United States (GSPC) market. A sliding window of 14 years and monthly intervals were used for the period spanning from 1950 to 2019.

For intuition on how the multifractal day-of-the-week effects change over time, we could analyze the time evolutions of the multifractal spectra. We considered the United States (GSPC) market, since the day-of-the-week effects over time here are well known [48]. For each day-of-the-week return, we constructed a sliding window of a size w=730 days with a sliding step Δ=5 days, meaning that we applied the MF-DFA method over a 14-year period in monthly intervals. Figure 5 illustrates the time evolutions of the multifractal spectra for different day-resolved returns. We observed that the spectrum evolved differently for each day of the week. For the Monday returns, the spectrum shifted to the left, which means that the fluctuations became less persistent over time. Other day-of-the-week returns either exhibited small movements in the multifractal spectra or moved back to the same position after some time. For a more quantitative analysis, we calculated the differences over time in the complexity parameters, namely $\Delta \alpha_{0}$ and ΔW, between Monday and other day-resolved returns. Figure 6a reveals that the spectra position of the Monday returns differed considerably from $\alpha_{0}$ of the other day returns in the first 15 years of the recorded period, but their differences dropped to zero in the subsequent years. This indicates the presence of strong day-of-the-week effects between 1950 and 1980 ($\Delta \alpha_{0}\to 0$ after 1965, where 1980 is already included because of the 14-year long sliding window), which is consistent with the literature, where it was found that the day-of-the-week effects diminished around 1980 [48].

Fluctuations around $\Delta \alpha_{0}=0$ after 1980 can be attributed to large financial crises that affected the entire market, such as Black Monday in 1987 and the global financial crisis in 2008. Figure 6b illustrates the time evolutions of the differences in the spectra width ΔW between Monday and other day-resolved returns. We observed that the Monday returns exhibited much wider multifractal spectra than other day’s returns during either of the two financial crises in 1987 and 2008. The Monday returns were characterized by more complex structures and had significant day-of-the-week effects during the financial crises even after 1980, when the effects from the calendar anomalies should have vanished. A possible explanation for this phenomena is the weekend gap in trading hours, which leads to even more speculative behavior from investors during a crisis.

## 5. Conclusions

This paper investigated the multifractal behavior of the day-of-the-week returns for market indices worldwide. We applied the MF-DFA method to daily returns for each day of the week (Monday returns, Tuesday returns and so on) and calculated the multifractal spectra as well as their complexity parameters. Considering the multifractal parameters’ positions of the maximum $\alpha_{0}$ and width W of an f(α) spectrum, we observed that distinct multifractal properties were found for the different days of the week, where North American, European and some Asian (South Korea, Indonesia and Hong Kong) and Latin American markets (Chile and Mexico) tended to show both stronger persistency ($\alpha_{0}>0.5$) and stronger multifractality (larger W) for the Monday returns, while for Australia, Indonesia and Taiwan, this tendency was found for the Tuesday returns. This finding agrees with the literature in that different day-of-the-week effects exist for different markets [25]. Some Asian markets displayed the Tuesday anomaly, being one day out of phase with the North American markets due to different time zones [64]. We found that multifractality arose from a broad probability density function and long-term correlations by analyzing shuffled series. The time-dependent multifractal analysis of the United States (GSPC) market revealed that the multifractal spectra for the Monday returns shifted to the left, or the fluctuations became less persistent over time. Other day-of-the-week returns exhibited small movements in the multifractal spectra. While the authors of [48] found that the effects from calendar anomalies vanished after 1980, in our study, we observed that the day-of-the-week effects persisted after the 1980s. Notably, the Monday returns exhibited much broader multifractal spectra compared with other days of the week. This behavior was especially pronounced around Black Monday on 19 October 1987 and the global financial crisis in 2008. A possible explanation for this phenomenon is the weekend gap in trading hours, leading to even more speculative behavior from investors during a crisis. Monday returns in general in the US tend to be different compared with those of other days of the week. This anomaly has been attributed to companies’ release of news after the financial markets close on Friday, and hence, the Monday prices reflect the accumulated reaction of investors over the weekend. This unique behavior of financial asset prices on Monday can be informative and useful for investment decision making and can inform policymakers to possibly limit important news releases on Friday afternoon. The Monday effect may be reduced by current tendencies of after-hours trading. However, since the after-hours trading volumes are much lower than the regular trading hours, the Monday effect is still present. Future studies should further investigate the multifractal dynamics and day-of-the-week effects for other financial markets and extend the current analysis to other calendar anomalies.

## Figures and Tables

**Table 1:** Information on analyzed time series for major market indices.

Market	Country	Index	Period
All Ordinares	Australia	AORD	3 August 1984–26 December 2018
S&P500/ASX 200	Australia	AXJO	22 November 1992–26 December 2018
BEL 20	Belgium	BFX	9 April 1991–24 December 2018
IBOVESPA	Brazil	BVSP	27 April 1993–21 December 2018
Dow30	United States	DJI	29 January 1985–26 December 2018
CAC 40	France	FCHI	1 March 1990–24 December 2018
DAX Performance	Germany	GDAXI	30 December 1987–27 December 2018
S&P500	United States	GSPC	3 January 1950–24 December 2018
S&P/TSX Composite	Canada	GSPTSE	29 June 1979–24 December 2018
Hang Seng Index	Hong Kong	HIS	31 December 1986–27 December 2018
IPSA Santiago de Chile	Chile	IPSA	2 January 2002–26 December 2018
Nasdaq	United States	IXIC	5 February 1971–26 December 2018
Jakarta Composite	Indonesia	JKSE	1 July 1997–27 December 2018
KOSPI Composite	South Korea	KS11	1 July 1997–26 December 2018
Merval	Argentina	MERV	8 October 1996–26 December 2018
IPC Mexico	Mexico	MXX	8 November 1991–26 December 2018
Nikkei 225	Japan	N225	5 January 1965–27 December 2018
NYSE Composite	United States	NYA	31 December 1965–26 December 2018
TSEC Weighted	Taiwan	TWII	2 July 1997–27 December 2018

**Table 2:** Multifractal parameters $\alpha_{0}$, *W* and *r* for day-resolved price returns $R^{i}$ of major market indices.

Market	Monday			Tuesday			Wednesday			Thursday			Friday			All		
	$\alpha_{0}$	W	r	$\alpha_{0}$	W	r	$\alpha_{0}$	W	r	$\alpha_{0}$	W	r	$\alpha_{0}$	W	r	$\alpha_{0}$	W	r
AORD	0.547	0.570	0.837	0.585	0.684	0.590	0.547	0.628	0.815	0.549	0.549	0.963	0.574	0.603	0.771	0.583	0.579	0.942
AXJO	0.537	0.529	0.990	0.583	0.514	1.201	0.561	0.558	0.897	0.557	0.544	1.180	0.562	0.550	0.866	0.533	0.748	0.913
BFX	0.619	0.633	0.730	0.561	0.662	1.383	0.571	0.541	0.754	0.574	0.553	0.940	0.553	0.556	0.715	0.534	0.676	1.188
BVSP	0.616	0.581	1.562	0.601	0.472	0.932	0.587	0.455	1.492	0.615	0.465	0.939	0.592	0.666	0.943	0.550	0.643	0.917
DJI	0.572	0.826	0.579	0.598	0.643	0.883	0.576	0.586	0.970	0.560	0.661	0.887	0.581	0.669	1.230	0.520	0.690	0.720
FCHI	0.617	0.656	0.969	0.526	0.621	1.257	0.579	0.613	1.087	0.620	0.579	1.090	0.553	0.535	0.894	0.506	0.633	1.174
GDAXI	0.606	0.612	0.682	0.556	0.619	0.886	0.574	0.530	0.986	0.616	0.538	1.034	0.555	0.485	1.397	0.534	0.648	1.176
GSPC	0.590	0.787	0.709	0.565	0.539	0.856	0.557	0.635	0.760	0.528	0.573	0.718	0.551	0.627	1.416	0.528	0.605	0.782
GSPTSE	0.611	0.632	0.683	0.587	0.647	0.841	0.581	0.618	0.956	0.587	0.552	0.733	0.554	0.681	0.775	0.585	0.613	0.928
HIS	0.582	0.823	0.828	0.562	0.669	0.639	0.514	0.730	0.864	0.592	0.509	1.083	0.576	0.749	0.878	0.557	0.609	0.805
IPSA	0.654	0.969	0.832	0.584	0.747	0.984	0.580	0.519	1.250	0.582	0.705	0.938	0.611	0.677	1.174	0.601	0.825	0.801
IXIC	0.641	0.707	0.764	0.585	0.644	0.941	0.615	0.702	0.781	0.563	0.671	1.425	0.587	0.680	1.134	0.591	0.624	0.901
JKSE	0.598	0.848	1.352	0.539	0.674	1.335	0.582	0.725	0.877	0.560	0.566	1.802	0.500	0.907	0.881	0.570	0.518	0.769
KS11	0.607	0.707	1.190	0.539	0.421	1.140	0.540	0.637	1.195	0.590	0.535	1.026	0.526	0.616	2.180	0.530	0.633	0.945
MERV	0.651	0.520	0.927	0.537	0.625	1.265	0.537	0.681	1.163	0.611	0.647	0.652	0.540	0.602	1.135	0.574	0.534	0.985
MXX	0.580	0.805	0.890	0.542	0.666	1.088	0.548	0.577	1.039	0.606	0.690	1.150	0.552	0.557	0.967	0.548	0.617	0.951
N225	0.584	0.472	1.041	0.573	0.745	0.714	0.550	0.639	0.901	0.614	0.505	0.732	0.553	0.530	0.804	0.539	0.406	0.559
NYA	0.593	0.685	0.466	0.579	0.648	0.790	0.550	0.588	0.615	0.559	0.691	0.827	0.526	0.573	0.954	0.522	0.583	0.772
TWII	0.659	0.474	1.661	0.594	0.564	1.453	0.519	0.453	1.069	0.540	0.494	1.584	0.503	0.764	1.303	0.539	0.491	1.053

**Table 3:** Differences in multifractal parameters between original and shuffled day-resolved price returns.

Market	Monday		Tuesday		Wednesday		Thursday		Friday	
	$\Delta \alpha_{0}$	ΔW	$\Delta \alpha_{0}$	ΔW	$\Delta \alpha_{0}$	ΔW	$\Delta \alpha_{0}$	ΔW	$\Delta \alpha_{0}$	ΔW
GSPC	0.049	0.115	0.030	0.019	0.021	0.094	0.008	0.058	0.014	0.126
KS11	0.033	0.010	0.034	0.219	0.028	0.052	0.012	0.138	0.044	0.052
IPSA	0.073	0.200	0.022	0.119	0.028	0.069	0.023	0.064	0.051	0.098
FCHI	0.064	0.035	0.023	0.078	0.031	0.054	0.066	0.033	0.002	0.025
